# Gallstone ileus 16 years postcholecystectomy?

**DOI:** 10.1016/j.radcr.2025.07.060

**Published:** 2025-08-22

**Authors:** Roma Tarar, Anishur Rahman

**Affiliations:** a New York Institute of Technology College of Osteopathic Medicine, 100 Northern Blvd Glen Head, Old Westbury, NY 11542, USA; b UHS Wilson Medical Center, 33-57 Harrison St, Johnson City, NY 13790, USA

**Keywords:** Gallstone ileus, Postcholecystectomy syndrome, Small-bowel obstruction, Long cystic duct, Cystic duct stone, Cholecystitis, Case report

## Abstract

Gallstone ileus, a rare complication of cholecystitis, occurs when the distal ileum becomes obstructed by gallstone causing, mechanical bowel obstruction. Gallstone ileus with history of cholecystectomy is exceptionally rare, with few cases documented in literature. This case report details the discovery of a large, impacted gallstone 16 years postcholecystectomy. An 87-year-old male presented with progressively worsening right-sided abdominal pain over 2-3 days. Exploratory laparotomy unveiled a 2.0 cm × 2.5 cm gallstone was obstructing the terminal ileum. Patient improved remarkably postoperatively and was discharged with resolution of symptoms. A potential cause for this large gallstone impaction within the terminal ileum is gallstone ileus, which can be explained with history of intentional or unintentional subtotal cholecystectomy or long cystic duct remnant. Both rare occurrences that allow gallstone formation or passage through a chronic cholecystoduodenal fistula due to inflammatory changes. With increasing trend toward subtotal cholecystectomies and division of cystic duct closer to the gallbladder neck, this case could potentially be explained as a gallstone ileus, despite extended postoperative intervals.

## Introduction

Gallstone disease, or cholelithiasis, is a prevalent cause of abdominal pain that, when severe, necessitates hospitalization. Despite being present in up to 15% of the North American population, only about 20% of this population develops symptoms [1]. Gallstones, most frequently composed of cholesterol and bilirubin, form within the gallbladder can obstruct the flow of bile into the duodenum. When blockage of the cystic or common bile duct occurs, bile can no longer transverse into the intestines, leading to reduced fat emulsification, poor absorption of such fats, and increased fat excreting into the stool. Consequently, undigested fat within the intestinal lumen has a lower density than normally digested chyme. This can cause fatty deposits on computed tomography (CT) and magnetic resonance cholangiopancreatography (MRCP) scans, steatorrhea, and altered fat-soluble vitamin absorption [2]. Symptomatic gallstones are typically sufficient to warrant cholecystectomy, which are largely performed laparoscopically. Laparoscopic cholecystectomy is hence the standard of care for symptomatic cholelithiasis with a relatively low complication rate [1].

Gallstone ileus is a rare but serious complication of cholelithiasis that occurs when a stone traverses a cholecystoduodenal fistula, leading to impaction with the ileum, ultimately causing a mechanical bowel obstruction. While this is a rare complication of cholelithiasis, it is even more uncommon many years after cholecystectomy, documented in only 0.4% of cases [3,4]. Risk factors for developing gallstone ileus include advanced age (60-70 years), female sex, long-standing cholelithiasis, recurrent cholecystitis, large gallstones (>2 cm), comorbidities (including hypertension and diabetes), and a history of abdominal surgery [5]. A case report describes the delayed presentation of gallstone ileus years after cholecystectomy caused by a large gallstone lost during an emergent laparoscopic cholecystectomy. In this case, the calculus eroded the bowel wall, causing recurrent sub-occlusive episodes [6].

Other potential explanations can be the formation of a fistula between remnant gallbladder and duodenum, incomplete removal of gallbladder (subtotal cholecystectomy), and long cystic duct [7,8]. Subtotal cholecystectomy may predispose patients to cholecystoduodenal fistulas and subsequent complications, including gallstone ileus.

This case describes an 87-year-old male that initially presented with small bowel obstruction that was managed conservatively and discharged home, but readmitted with recurrent symptoms and CT evidence of bowel obstruction. Due to recurrent bowel obstruction, he required exploratory laparotomy and was found to have a very large, impacted gallstone within the terminal ileum. Our description has been reported in line with the SCARE criteria for case reporting [9].

## Case presentation

An 87-year-old Caucasian male with medical history of hypertension, alcoholism, dyslipidemia, heart failure, persistent atrial fibrillation on apixaban, gout, and prostate cancer (s/p radiation in 2001), presented with worsening right-sided abdominal pain and distension. He had previously undergone laparoscopic cholecystectomy and bilateral laparoscopic inguinal hernia repairs; all performed 16 years prior. Operative notes for all prior surgeries could not be obtained; therefore, specific details about the procedure were unavailable. Additionally, patient could not recall any information regarding the procedures. Family history is noncontributory. Home medications include apixaban, atorvastatin, carvedilol, furosemide, and valsartan. He was a former smoker and admitted to consuming alcohol for approximately 62 years, beginning around the age of 25. Patient typically consumed spirits, averaging 5-8 drinks per day, with several failed attempts to reduce use.

**Fig. 1:**
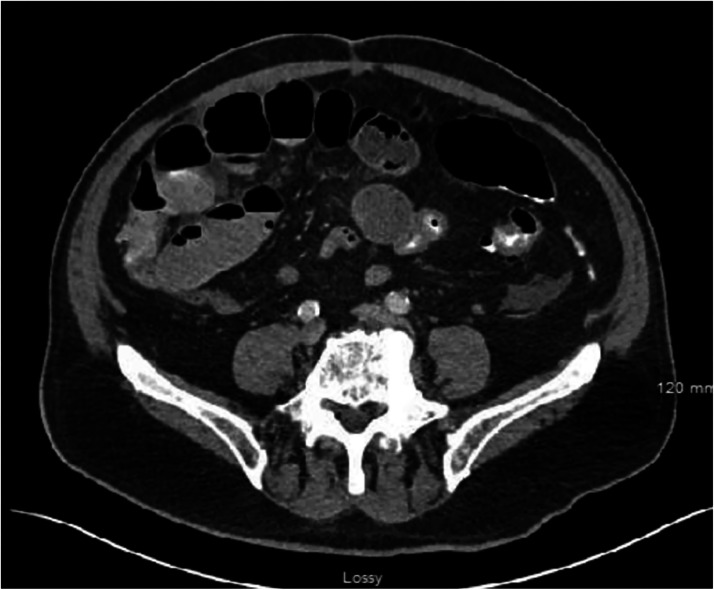
Axial abdominal CT image on admission displays findings including dilated small bowel and air-fluid levels consistent with partial bowel obstruction.

Patient presented to the emergency department due to right-sided abdominal pain and distension progressing over 2-3 days that suddenly worsened overnight. His last bowel movement was 4 days prior. Patient reported that he had been having abdominal discomfort for many months, having had three hospital admissions in the preceding 7 months. Initial clinical exam findings included abdominal distension, diminished bowel sounds, and generalized tenderness. Objective laboratory findings showed no leukocytosis, liver function test within normal limits, as well as lipase and lactate within range. Remainder of metabolic panel, including electrolytes were within normal limits. CT of abdomen and pelvis showed partial bowel obstruction and confirmed status post cholecystectomy with presence of clips and compensatory dilation of the common bile duct (Fig. 1). Liver showed mild fatty infiltration with no masses. Initially, he was placed on nothing by mouth (n.p.o.), intravenous (IV) hydration, and treated with ondansetron and pain control as needed. Nasogastric (NG) tube was attempted but not successful. Despite some gas passage, bowel movements were absent. General surgery was consulted, and he underwent Gastrografin small bowel series study 2 days after admission, that showed no evidence for complete small bowel obstruction. Patient post-Gastrografin study reported bowel movement and continuous passing of gas. He was started on clear liquid diet followed by advancement to regular diet the next day, both of which he tolerated well. Patient was discharged on day 3 following regular bowel movement and toleration of regular diet.

**Fig. 2:**
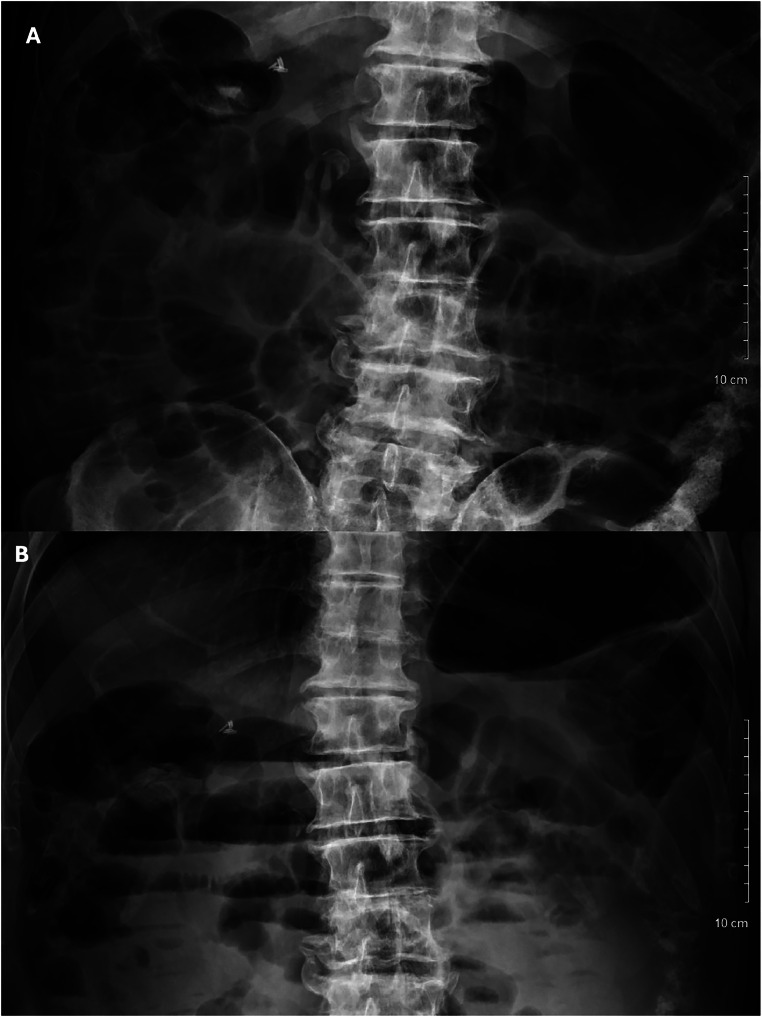
Supine (A) and upright (B) anteroposterior abdominal radiograph upon readmission showing gaseous distention of multiple loops of small bowel with air-fluid levels.

**Fig. 3:**
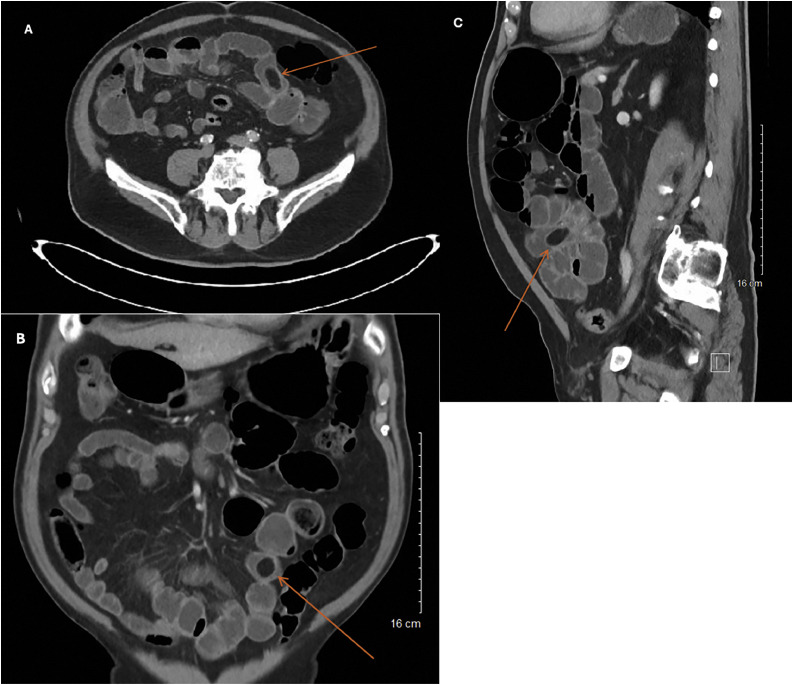
Axial (A), coronal (B), and sagittal (C) abdominal CT images on readmission, significant for filling defect consistent with an obstructive agent (orange arrow).

The next day, the patient returned to the emergency department with worsening abdominal pain, distension, heartburn, and some flatulence. He admitted to of one episode of diarrhea the day prior after discharge, but denied additional bowel movements since then. He had not been consuming food and denied vomiting. Abdominal radiograph upon re-admission showed gaseous distension of multiple loops of small bowel with air-fluid levels along with gaseous distention of the colon although no gross free intraperitoneal air was detected (Fig. 2). CT scan of abdomen and pelvis also consistent with distal small bowel obstruction (Fig. 3). WBC was elevated with left shift at 13.3 cells/mL. C-reactive protein was 7.2 mg/L, glucose was 130 mmol/L, and alkaline phosphate was 88 U/L. Otherwise, metabolic panel was within normal limits, as seen in Table 1. Patient was placed on n.p.o., IV normal saline, IV pantoprazole, and ondansetron. General surgery consultation prompted decision to proceed with exploratory laparotomy due to recurrent bowel obstruction.

**Table 1:** Preoperative and postoperative objective laboratory values.

Objective laboratory values	Preoperative values	Postoperative values	Reference range
Sodium (mEq/L)	139	137	136-146
Potassium (mEq/L)	4.0	3.8	3.5-5.0
Chloride (mEq/L)	101	104	95-105
Bicarbonate (mEq/L)	27	25	22-28
Calcium (mg/dL)	9.3	8.0	8.4-10.2
Albumin (g/dL)	4.5	3.2	3.5-5.5
Alkaline phosphate (U/L)	88	187	25-100
Alanine aminotransferase (ALT) (U/L)	25	55	10-40
Aspartate aminotransferase (AST) (U/L)	45	59	12-38
Bilirubin (total) (mg/dL)	0.9	1.0	0.1-1.0
Hematocrit (%)	45.9	38.5	41-53
Hemoglobin (g/dL)	15.6	12.8	13.5-17.5
Leukocyte count (WBC) (mm^3^)	13.3	10.2	4.5-11.0
RBC (10^6^/µL)	4.00	4.86	4.2-5.5
Platelet count (mm^3^)	275	180	150-400
Prothrombin time (PT)	20.8	17.2	11-15
INR	1.80	1.52	<1
Glucose (mg/dL)	130	95	70-110
C-reactive protein (mg/L)	7.2	4.1	<1.0

Patient was prepared for exploratory laparotomy on day 2 of readmission under general anesthesia with placement of NG tube. Midline incision (8 cm) was made from supraumbilical to infraumbilical area with depth into the peritoneal cavity. In entering the abdominal cavity, liver bed was inspected and found to be smooth, uniform in color and texture, with no signs of abnormality or bleeding. Intraoperatively, proximal small intestine was found to be significantly distended with decompression of the ileum beyond large 2.0 cm × 2.5 cm impacted gallstone. The stone was milked proximally to the jejunum, a longitudinal 2.5 cm incision was made at the antimesenteric border, and the impacted large gallstone was removed and sent to pathology. Enterotomy opening was approximated with 3.0 Vicryl suture in a Hienicke-Mickulitz technique and reinforced with 3-0 silk sutures in an interrupted imbricating fashion. Abdominal cavity was irrigated, and fluid was suctioned. Patient tolerated the procedure well. Pathology report described large yellow-tan gallstone measuring 2.0 cm × 2.5 cm. Histological examination was not performed as it is not routine for gallstones, as the vast majority of these specimens show benign pathology.

Postoperatively, he was then placed on n.p.o., NG tube remained in place, and started on total parenteral nutrition along with IV fluids. Patient developed persistent ileus postoperatively, and repeat abdominal x-ray on postoperative day 6 showed air within multiple nonpathological loops of bowel. Small bowel series with Gastrografin on postoperative day 7 showed residual contrast in the right colon and mild nonspecific gaseous distention of small bowel loops without transition. Contrast was seen distal to the ileum by 2 hours and within left colon at 4 hours, indicating no obstruction. On postoperative day 8, patient had bowel movements, NG tube was removed, diet was advanced to clear liquids, and total parenteral nutrition was weaned. Patient tolerated clear liquids and was advanced to regular diet on postoperative day 9. Patient was medically stable at this point and discharged the following day. Patient, evaluated 7 days after discharge at follow-up visit, was found to be recovering well with no complications and satisfactory healing progress.

## Discussion

This case exemplifies an exceptionally uncommon instance of gallstone ileus 16 years after laparoscopic cholecystectomy. The patient initially presented with partial small bowel obstruction after prolonged periodic episodes of abdominal pain, bloating, and constipation. After ruling out complete bowel obstruction on small bowel series and toleration of oral liquid diet, the patient was discharged, only for him to return the following day with signs of complete bowel obstruction. This is likely due to progression of the impacted gallstone within the terminal ileum, causing complete obstruction. Additionally, the time interval between the initial laparoscopic cholecystectomy and gallstone ileus presentation is atypical [10]. Most commonly in patients with prolonged cholelithiasis, gallstone ileus is a potential explanation of small bowel obstruction [11]. As in our case, the delayed presentation of large obstructing gallstone years after cholecystectomy came with diagnostic uncertainty and was followed with important deliberation regarding pathogenesis.

As in this case, imaging findings for gallstone ileus can present subtlety, leading to missed or delayed diagnosis and prolonged hospital course. In retrospective review of the CT scan from repeat admission, a filling defect within the terminal ileum was identified, corresponding to the causative agent of the bowel obstruction, which was the gallstone (Fig. 3). Gallstones can be classified as cholesterol, pigment, or mixed stones. Cholesterol stones are typically hypoattenuating on CT scans, as seen in our patient’s imaging. Pigment stones, composed of bilirubin and calcium salts, are more commonly hyperattenuating, while mixed stones have variable appearances [12]. Distinguishing stone attenuation and consequently composition on imaging serves as an aid in identifying the etiology of obstructing stones. During the initial presentation, the absence of liver function test abnormalities along with the remote history of cholecystectomy led to reasonable deprioritization of advanced biliary imaging, including MRCP or endoscopic retrograde cholangiopancreatography.

Delayed development of gallstone ileus after laparoscopic cholecystectomy is uncommon, particularly to the extent presented in our case [13]. Literature review presents a rare cause of bowel obstruction in patients with history of cholecystectomy occurring within months to a few years, being gallstone ileus, with precipitating factors including retained stones and the formation of fistula [[14], [15], [16]]. One potential mechanism involves retained gallstones due to subtotal cholecystectomy or long cystic duct remanent, both scenarios of which can predispose to fistula formation and subsequent impaction within the distal ileum, causing small bowel obstruction [17]. This anatomical presentation allows for recurrent inflammation and eventual formation of a cholecystoduodenal fistula with which a stone can migrate into the bowel lumen. As seen in the table adapted from Nuño-Guzmán et al. (Table 2), cholecysto-duodenal fistulas are the most common biliary-enteric fistulas in gallstone ileus cases [10]. The most common locations for obstruction to develop as a result of these fistulas is the terminal ileum in about 70% of cases [17].

**Table 2:** **Incidence of biliary-enteric fistulas in gallstone ileus cases, as adapted and modified from Nuño-Guzmán et al. [****10****]**.

Type of fistula	Range (%)
Cholecystoduodenal	32.5-96.5
Cholecystogastric	0-13.3
Cholecystojejunal	0-2.5
Choledochoduodenal	0-13.4
Undetermined	0-65

Given the prolonged asymptomatic period between the patient’s gallbladder removal and presentation, the stone was not likely to have been present within the terminal ileum throughout the patient’s asymptomatic years. Instead, this could be explained by a temporary sequestration of the stone within a secondary location in a duodenal or proximal jejunal diverticulum, eventually dislodging and impacting within the terminal ileum, causing bowel obstruction [18]. Diverticula can occasionally serve as sites of temporary stone sequestration, commonly occurring when stones enter the bowel via a biliary-enteric fistula [18]. Over time, changes within peristalsis, progression of diverticular disease, and local inflammation can lead to the stone dislodging and migration to narrow segments of the bowel, typically the terminal ileum. The stone can become impacted, causing a mechanical obstruction and ultimately gallstone ileus [19]. This delayed impaction could mimic our case presentation; however, confirmation of such an occurrence is exceedingly challenging without intraoperative or histological evidence, not found in our case.

An additional potential explanation that was considered was the presence of remanent gallbladder and long cystic duct, which could have served as a reservoir for retained or newly formed gallstones [20]. The presentation of a gallstone years after cholecystectomy is possible in this case, as duct patency and communication of the biliary system would explain a delayed presentation as biliary stasis contributing to gallstone formation over time [20]. In any case, remanent gallbladder and long cystic duct could potentially be identified on imaging modalities, including ultrasound, CT as well as MRCP and endoscopic retrograde cholangiopancreatography, not performed in our case presentation. The possibility of choledochoduodenal fistula with primary choledocholithiasis was also considered but deemed unlikely given the large size (∼2 cm in length) of the obstructing gallstone within the common bile duct [21]. The formation of stones within the bile duct itself, also known as primary choledocholithiasis, would be unlikely in this case as it would rarely progress to this magnitude without causing symptoms earlier [21]. In contrast, primary choledocholithiasis is seen more commonly in patients with choledochal strictures or cysts leading to bile stasis, which was not apparent in our patient [22].

Differential diagnosis for patients presenting with symptoms of small bowel obstruction with a remote history of cholecystectomy should include delayed biliary complications, especially in cases with subtotal cholecystectomy. Subtotal cholecystectomy is an increasingly favored procedure in challenging operative fields, severe inflammation, or difficult anatomy to avoid injury to the common bile duct and other nearby structures [23]. Subtotal cholecystectomy involving division of the cystic duct closer to the gallbladder or leaving a remanent structure in place inadvertently predisposes patients to persistent symptoms, inflammation, and gallstone-related complications even years later [[24], [25], [26]]. A recent rise in subtotal procedures is likely due to increased use of laparoscopic approach, prevention of bile duct injury, and the option as an alternative to total cholecystectomy in high-risk patients [27]. Two common approaches are the fenestrating open approach and the reconstituting approach. Fenestrating subtotal cholecystectomy involves a patent cystic duct, increasing the risk of bile leaks, while the reconstituting approach closes the cystic duct with sutures/staples, with an increased risk of retained stones [28]. Additional complications include remnant gallbladder or cystic duct stump, which can act as a nidus for stone formation or harbor residual stones [20]. Prolonged time and progressive inflammation can precipitate fistula formation and gallstone ileus [29]. Awareness of long-term sequelae has become increasingly important as subtotal cholecystectomy has become more common [30].

Gallstone ileus in relatively uncommon, but when present, is a significantly implicated in small bowel obstruction [31]. This is most seen in patients with an intact gallbladder but has rarely been reported years after cholecystectomy [31]. These rare cases are more likely seen in patients with gallbladder remnant or persistent long cystic ducts. The atypical presentation and history of cholecystectomy can delay diagnosis and treatment [32]. Gallstone ileus is diagnosed conclusively with imaging, including CT scan, revealing the classic Rigler’s triad: pneumobilia, small bowel obstruction, and ectopic gallstone [33]. In patients with history of cholecystectomy, presence of a biliary-enteric fistula or patent remnant cystic duct can be overlooked on diagnostic imaging. Managing gallstone ileus in such patients is largely based on the patient’s clinical stability, severity of symptoms, and operative comorbidities [34]. Definitive treatment aims to relieve the obstruction with options including enterolithotomy alone, enterolithotomy with remanent cholecystectomy, and fistula closure in a one or two-stage approach [35]. Alternatively, in high-risk patients with partial or mobile obstruction, nonoperative or minimally invasive approaches may be preferred. Postoperative monitoring for biliary leaks and recurrent fistula formation is essential to limit morbidity [36].

## Conclusion

This case highlights a rare yet significant complication of laparoscopic cholecystectomy, emphasizing the importance of considering biliary ramifications even decades after gallbladder removal. It highlights the role of diagnostic imaging and evaluation when considering residual biliary anatomy, stone retention, and fistula formation many years after gallbladder removal. With rising subtotal cholecystectomy procedures, maintaining awareness and prolonged surveillance of potential complications such as gallstone ileus is critical.

## Patient consent

Written informed consent was obtained from the patient for publication and any accompanying images. A copy of the written consent is available for review by the Editor-in-Chief of this journal on request.
